# *Bacillus velezensis* 7-A as a Biocontrol Agent Against *Fusarium verticillioides*, the Causal Agent of Rice Sheath Rot Disease

**DOI:** 10.3390/microorganisms13112511

**Published:** 2025-10-31

**Authors:** Boyu Liu, Qunying Qin, Jianchao Hu, Jiayi Wang, Juan Gan, Ye Zhuang, Zhengxiang Sun, Yi Zhou

**Affiliations:** 1 College of Agriculture, Yangtze University, Jingzhou 434025, China; 15271728234@163.com (B.L.); qqy123452025@126.com (Q.Q.); 15067920433@163.com (J.H.); 13264675061@163.com (J.W.); 13595777617@163.com (J.G.); yvhexiongzhang@163.com (Y.Z.); 2 MARA Key Laboratory of Sustainable Crop Production in the Middle Reaches of the Yangtze River (Co-Construction by Ministry and Province), Hubei Key Laboratory of Waterlogging Disaster and Agricultural Use of Wetland, College of Agriculture, Yangtze University, Jingzhou 434025, China

**Keywords:** *Fusarium verticillioides*, *Bacillus velezensis*, biological control, secondary metabolites, rice sheath rot, disease resistance mechanisms

## Abstract

Rice sheath rot has progressively developed into a growing threat to global rice production, particularly in intensively managed systems conducive to disease development. Therefore, accurate identification of the causal pathogen and the development of sustainable management strategies represent urgent scientific requirements. In this study, we isolated the causal organism of rice sheath rot from infected rice tissues and identified it as *Fusarium verticillioides* based on multi-locus sequence analysis. Eight endophytic bacterial strains were recovered from healthy rice root systems. Among the isolates, *Bacillus velezensis* isolate 7-A exhibited the strongest antifungal activity against *F. verticillioides*. This isolate demonstrated broad-spectrum antifungal activity, with inhibition rates ranging from 54.8% to 71.8%. Phylogenetic analysis based on 16S rRNA and *gyrB* gene sequences identified it as *B. velezensis*. Further characterization revealed that *B. velezensis* 7-A is capable of secreting proteases and synthesizing siderophores. The filtered liquid from sterile fermentation markedly inhibited the growth of mycelium in *F. verticillioides* and induced marked morphological abnormalities. Liquid LC-MS analysis identified multiple antifungal active substances, including camphor, ginkgolides B, salicin, cinnamic acid, hydroxygenkwanin, stearamide, *β*-carotene, and others. A pot experiment demonstrated that the fermentation broth of *B. velezensis* 7-A effectively suppressed the occurrence of rice sheath rot, achieving a relative control efficacy of 61.3%, which is comparable to that of a 10% carbendazim water-dispersible granule (WDG). Additionally, isolate 7-A enhances plant disease resistance by activating the activities of key defense enzymes. These findings provide preliminary insights into its potential application in integrated and sustainable disease management programs.

## 1. Introduction

Rice sheath rot is a fungal disease that threatens global rice production. It primarily affects the flag leaf sheaths during critical growth stages, especially around heading, and can cause severe yield losses under favorable environmental conditions. The causal agent is thermophilic and hygrophilic, and its epidemic development is closely related to the ambient temperature and humidity during the reproductive stage of rice growth [1]. Field observations indicate rapid disease progression, with symptoms typically developing into characteristic lesions within 48–72 h after infection. The pathogen colonizes the lower leaf sheaths and subsequently spreads to the panicle through internal translocation during the heading stage. Disease severity depends on rice cultivars; slow-heading varieties are much more susceptible than fast-heading types [2]. The major feature of rice sheath rot is rotting and discoloration of the sheath [3]. Sheath rot infection leads to chaffy, discolored grains and affects the viability and nutritional value of seeds [1,4].

In recent years, rice sheath rot has become more prevalent in major rice-growing regions in China, along with the changes in agriculture and ecosystem dynamics, including climate warming, high-density planting, and excessive nitrogen [5], but the disease etiology and pathogen biology remain poorly understood. Historically, *Sarocladium oryzae* (syn. *Acrocylindrium oryzae*) was first identified as the primary causal agent of rice sheath rot in Taiwan in 1922 [6]. Subsequent taxonomic revisions have recognized additional species within the genus *Sarocladium* [3], including *S. attenuatum* and *S. sparsum*, which are now reported from 38 rice-producing countries worldwide [7]. Additionally, recent studies have revealed a more complex etiological scenario, with co-occurrence of other pathogens such as *Fusarium* spp., particularly members of the *F. fujikuroi* species complex [8,9,10], and *Pseudomonas fuscovaginae* in disease complexes [11]; although a few studies have suggested the involvement of *F. verticillioides* in rice sheath rot [3], it is not considered a primary causal pathogen of the disease under field conditions. This species is more commonly recognized as a major pathogen of maize and other crops [12]. Concerningly, *Fusarium* pathogens not only cause substantial crop losses, but also produce harmful mycotoxins, such as fumonisins and trichothecenes, which contaminate grain and pose serious risks to human and animal health through the food chain [13,14].

Over the past decade, several systematic disease management strategies have been used to control this disease, including resistant cultivars, chemical pesticides [15,16], crop rotation, and soil amendments [17,18], but these approaches have significant drawbacks: long-term use of synthetic fungicides can lead to pathogen resistance, resistance, decreased variety of soil microbes, soil microbial diversity, and environmental pollution [19,20]. Recent research indicates that microorganism-mediated biocontrol agents (BCAs) can help control plant and soilborne diseases [21], reducing the incidence of soilborne and foliar plant disease, e.g., through competitive inhibition, production of antimicrobial compounds, host resistance, and alteration of the microbial community structure [22,23].

*B. velezensis* has emerged as a multifunctional biocontrol agent that demonstrates direct antagonism by producing antimicrobial metabolites such as lipopeptides and polyketides, systemic resistance (ISR) in host plants, and plant growth promoted through phytohormone modulation [24]. For example, Myo et al. [25] demonstrated that the volatile organic compounds (VOCs) generated by *B. velezensis* NKG-2 negatively affected the growth of several fungi. Meng et al. [26] reported that the B4-7 enhanced tobacco’s resistance to *R. solanacearum* by upregulating defense-related enzyme activities, including superoxide dismutase (SOD), phenylalanine ammonia-lyase (PAL), peroxidase (POD), catalase (CAT), and polyphenol oxidase (PPO). Although *Bacillus velezensis* 7-A has been shown to be effective against a variety of phytopathogens [27,28], its application in controlling rice sheath rot is still unexplored.

In this study, an uncommon pathogen associated with rice sheath rot was successfully isolated and characterized. A bacterial isolate exhibiting inhibitory effects against *F. verticillioides* was isolated and screened from the healthy rice root tissues. In addition, the isolate was identified, its antifungal activity was evaluated, and its control spectrum was determined. The underlying antifungal mechanism was further examined. This research offers novel microbial resources for the biological management of rice sheath rot, and also contributes to the advancement of sustainable agricultural practices by offering an environmentally friendly alternative to chemical control for the management of rice sheath rot. To the authors’ knowledge, this is the first report of the endophytic *B. velezensis* isolate isolated from *Oryza sativa* that exhibits strong antifungal activity against *F. verticillioides*.

## 2. Materials and Methods

### 2.1. Sample Collection and Processing

In September 2024, rice (*Oryza sativa* L. ‘Nipponbare’) plants exhibiting typical symptoms of sheath rot were collected from experimental paddy fields at the West Campus of Yangtze University (30°21′ N, 112°09′ E), Jingzhou City, Hubei Province, China. Symptomatic plants were selected based on well-defined diagnostic criteria, including the presence of characteristic necrotic lesions with water-soaked margins on the leaf sheaths. Diseased tissues were aseptically excised using sterilized scalpels, immediately placed in pre-sterilized sampling bags, and kept at 4 °C during transportation to the laboratory to prevent microbial contamination and preserve pathogen viability. Each sample was labeled with detailed field information, including GPS coordinates, collection date, and symptom severity index, prior to further processing for pathogen isolation and identification.

### 2.2. Pathogen Isolation

Diseased tissue samples were excised into small segments (5 mm × 5 mm) and subjected to surface sterilization through sequential immersion in 70% ethanol for 30 s, followed by a 1% sodium hypochlorite solution for 1 min, then rinsed three times with sterile distilled water. The sterilized tissue fragments were then aseptically transferred onto potato dextrose agar (PDA) plates, with 3–5 segments per plate, and incubated at 25 °C for 5–7 d under dark conditions to facilitate fungal growth.

Fungal hyphal tips were carefully excised from the advancing margins of the colony and transferred onto fresh PDA plates using the sterile inoculating needle. The subculturing process was repeated 3 to 5 times until the colonies exhibited consistent and uniform morphological characteristics, ensuring the establishment of purified cultures [29].

### 2.3. Identification of Fusarium Species Based on Multi-Locus Sequence Analysis

Genomic DNA was extracted from each isolated *Fusarium* isolate using the DNeasy UltraClean Microbial Kit (Qiagen, Hilden, Germany) according to the manufacturer’s instructions. PCR amplification was then carried out for three gene regions as specified (Table 1), according to the manufacturer’s protocol. The amplified products were purified and submitted to Wuhan Qingke Biotechnology for Sanger sequencing.

Phylogenetic analysis was conducted using both sequences obtained in this study and reference sequences retrieved from the NCBI nucleotide database (GenBank). Initial sequence comparisons with the NCBI database enabled classification of the isolated *Fusarium* isolates into five distinct species complexes (referred to here as “series”). For phylogenetic reconstruction, individual trees were generated for each *Fusarium* series using a concatenated dataset comprising *ITS*, *TUB*, and *RPB2* gene sequences. Species identities and corresponding GenBank accession numbers are listed in Table 1. *F. nygamai* CBS 139,387 and *F. proliferatum* CBS 480.96 were selected as outgroup taxa for phylogenetic tree construction. All positions containing gaps or missing data were excluded from the analysis through pairwise deletion. The maximum likelihood (MP) tree was inferred using the subtree pruning and regrafting (SPR) algorithm implemented in MEGA7 following the methodology described by Engeset [30].

**Table 1:** Primer sequences and annealing temperatures used for PCR amplification of fungal genetic markers.

Gene Name	Primer Sequence	Annealing Temperature	Reference
*ITS*	F: TCCGTAGGTGAACCTGCGG R: TCCTCCGCTTATTGATATGC	55 °C	Innis et al., 2012 [31]
*RPB2*	F: GAYGAYCGKGAYCAYTTCGG R: CCCATRGCYTGYTTRCCCAT	50 °C	Xia et al., 2019 [32]
*TUB*	F: AACATGCGTGAGATTGTAAGT R: ACCCTCAGTGTAGTGACCCTTGGC	58 °C	Glass et al., 1995 [33]

Note: The forward (F) and reverse (R) primers target three standard genetic regions: the internal transcribed spacer (*ITS*), β-tubulin (*TUB*), and the second largest subunit of RNA polymerase II (*RPB2*). The F primer binds to the antisense strand and initiates 5′→3′ amplification along the sense strand, while the R primer binds to the sense strand and extends toward the 5′ end of the antisense strand. Annealing temperatures were optimized for each primer pair, and all sequences were provided in the 5′ to 3′ orientation.

### 2.4. Pathogenicity Assay

Rice (*sativa* ‘Nipponbare’) seeds were surface sterilized by sequential immersion in 75% ethanol for 1 min, followed by 2% sodium hypochlorite solution for 3 min, then rinsed three times with sterile distilled water. Seedlings were germinated on moistened sterile filter paper at 28 °C, and subsequently transplanted into pots. Plants were grown until they reached the heading stage, after which those exhibiting uniform development were selected for pathogenicity testing.

For the inoculation treatment, three plants per replicate were inoculated at the second sheath. A 7 mm mycelial plug was excised from the actively growing margin of a 5-day-old culture of the target pathogen on PDA and placed onto each wound site; artificial wounds were created on the flag leaf sheath using a sterile needle (0.1 mm diameter) to make two shallow, oblique punctures (2–3 mm depth, 5 mm apart) 2 cm from the stem, allowing pathogen entry while minimizing tissue damage. Control plants were treated identically with sterile PDA plugs without fungal inoculum. All inoculated plants were incubated in a controlled-environment growth chamber (Shanghai Yuejin Medical Instrument Factory, Shanghai, China) under standardized conditions: 25 ± 1 °C, 75% relative humidity, and a 14/10 h light/dark photoperiod. Disease progression was monitored daily, and representative symptoms were recorded at 7 d post-inoculation (dpi) using a standardized digital imaging system (Canon EOS 90D with macro lens, Canon Inc., Tokyo, Japan).

### 2.5. Isolation and Screening of Isolate 7-A

#### 2.5.1. Isolation of Endophytic Bacteria

Rice root samples were gathered from the West Campus of Yangtze University in Hubei Province, China; disinfected using a 3% NaClO solution for 6 min and 70% ethanol for 1 min; and rinsed 4–5 times with sterile distilled water [34]. The sterilization process effectiveness was tested by incubating (4 d at 37 °C) 100 mL of the last wash water on LB plates, and no bacterial growth was observed. Approximately 0.1 g of surface-sterilized plant tissue was placed in a mortar containing 1 mL of sterile phosphate-buffered saline (PBS, pH 7.4). The tissue was homogenized using a sterile pestle, and the resulting suspension was transferred to a 2 mL microcentrifuge tube. The homogenate was vortexed vigorously and then shaken at 200 rpm for 10 min to facilitate the release of endophytic microorganisms. The filtrate was collected and diluted to various concentrations (10-6, 10-7, and 10-8) with sterile water, then 20 μL of each dilution was spread onto LB agar plates for CFU counting. The medium contained (per liter) 10 g tryptone, 5 g yeast extract, 10 g NaCl, and 15 g bacteriological agar. The pH was adjusted to 7.0 with NaOH prior to sterilization by autoclaving at 121 °C for 20 min. Purified isolates were preserved in 15% glycerol solution and stored at −80 °C for long-term maintenance [35].

#### 2.5.2. Assessment of Antifungal Bacteria

The dual-culture assay was used to examine the ability of endophytic bacterial isolates to inhibit the growth of pathogens [36,37]. On each PDA plate, a hyphal plug of *F. verticillioides* (5 mm) was positioned, and a single, symmetrical hole (6 mm) was drilled 4 cm from the plug. The hole was filled with 100 μL of the endophytic bacterial fermentation broth and 100 μL of LB medium as the control (CK). The plates were incubated at 28 °C for 5–7 d, while for the control group the entire plate was covered with *F. verticillioides.* Based on the size of the inhibition zone, isolates exhibiting potent antifungal activity against *F. verticillioides* growth were chosen. To guarantee repeatability, the full experiment was conducted three times, with three replicate trials conducted for each treatment group [38].

Inhibition rate (%) = [(Colony diameter of control − Colony diameter of treatment)/(Colony diameter of control − Initial colony diameter)] × 100.

### 2.6. Identification of Isolate 7-A

Genomic DNA was extracted following the method of Tamura [39], and the 16S rRNA gene (16S rRNA) was specifically amplified via PCR using the universal primers 27F (5′–AGAGTTTGATCCTGGCTCAG–3′) and 1492R (5′–TACGGCTACCTTGTTACGACT–3′) [40]. The PCR mixture (20 μL) contained 1.5 μL of template DNA, 1 μL of each primer (50 pmol/μL), 1.5 μL of Taq DNA polymerase, 2 μL of Mg^2+^ solution (25 mmol/L), 2 μL of 10× buffer, 0.5 μL of dNTPs (10 mmol/L), and nuclease-free water to make up the final volume. The thermal cycling conditions were as follows: initial denaturation at 94 °C for 10 min, followed by 30 cycles of denaturation at 94 °C for 1 min, annealing at 55 °C for 1 min, and extension at 72 °C for 1 min, concluding with a final extension at 72 °C for 10 min. “A no-template control (NTC), in which sterile nuclease-free water replaced the DNA template, was included in each PCR assay to detect contamination. No amplification was observed in any NTC.” Specific amplification of the *gyrB* gene was performed according to the protocol described by Yamamoto, using the primers Up-1 (5′–GAAGTCATCATGACCGT TCTGCAYGCNGGNNGGNAARTTYGA–3′) and Up-2r (5′–AGCAGGGTACGGATGTGCGAGCCRTC–NACRTCNCRTCNGTCAT–3′) [41]. 

### 2.7. Determination of the Antifungal Spectrum of Isolate 7-A

The processes outlined in Section 2.5.2 were adhered to in the technique used in this investigation. Specifically, the mycelial growth inhibition rate of the most effective antifungal isolate 7-A was assessed against six pathogenic fungi: *Rigidoporus microporus* (red root rot of rubber tree), *F. oxysporum* (*Fusarium* wilt of eggplant), *Cochliobolus heterostrophus* (Maize Leaf Spot), *Rhizoctonia solani* (rice sheath blight), *Phytophthora nicotianae* (target spot in tobacco), and *Gibberella fujikuroi* (Bakanae disease of rice). All fungal isolates were deposited in the Fungal Herbarium of Yangtze University (Jingzhou, Hubei Province, China). To ensure reproducibility and data reliability, all experimental treatments were performed in triplicate.

### 2.8. Inhibition Assay of Bacterial Culture Filtrate (BCF) Fermentation Broth from Biocontrol Isolate 7-A

A single colony of isolate 7-A was inoculated into potato dextrose broth (PDB) and incubated in a rotary shaker at 27 ± 1 °C with agitation at 180 rpm for 3 d. Following incubation, the culture was centrifuged at 10,000× *g* for 15 min at 4 °C, and the supernatant was collected aseptically. The bacterial culture filtrate (BCF) was sterilized by filtration through a 0.22 μm pore-size membrane under laminar airflow and stored in sterile conical flasks at 4 °C until further use. To evaluate antifungal activity, potato dextrose agar (PDA) medium was supplemented with BCF to achieve final concentrations of 0% (control), 1%, 5%, and 10% (*v*/*v*), following the methods described by Lai [42]. Mycelial plugs (7 mm diameter) of *F*. *verticillioides* were obtained from the actively growing margins of 7-day-old cultures using a sterile cork borer and placed at the center of each PDA plate amended with BCF. Each treatment included three replicate plates. Following incubation at 27 ± 1 °C for 7 d in a growth chamber, the mycelial growth of *F. verticillioides* was assessed. Simultaneously, mycelia from colonies grown on plates supplemented with different concentrations of fermentation broth were examined microscopically to observe morphological structures and color changes. Microscopic examination was performed using an optical microscope (Olympus BX53, Tokyo, Japan) equipped with a digital camera (Olympus DP74, Tokyo, Japan), manufactured by Olympus Corporation. Images were captured at 400× magnification to document variations in hyphal morphology and pigmentation.

### 2.9. Pot Experiment Evaluating the Biocontrol Efficacy of Isolate 7-A Against Rice Sheath Rot

A pot experiment was conducted in a controlled-environment greenhouse (25 °C) at the College of Agriculture, Yangtze University, from October 2024 to February 2025, to evaluate the biocontrol potential of isolate 7-A against rice sheath rot caused by *F*. *verticillioides*. The experiment was arranged in a completely randomized design (CRD) with four treatments and three replicate pots per treatment (total of twelve pots): Negative control: plants inoculated solely with *F. verticillioides* without protective treatment; biocontrol control (no pathogen): plants treated with sterile suspension of isolate 7-A without subsequent pathogen challenge to evaluate plant responses to the bacterium alone; BCF treatment: plants pretreated with cell-free filtrate (BCF) from isolate 7-A culture followed by pathogen inoculation; and positive control: plants pretreated with 10% carbendazim water-dispersible granule (WDG, 1000-fold dilution) prior to pathogen inoculation. In all protection groups (BCF and positive control), treatments were administered via root drenching 24 h before pathogen inoculation to assess induced resistance or preventive antifungal effects. For BCF preparation, the bacterial culture was centrifuged (8000× *g*, 10 min) and the supernatant was filter-sterilized (0.22 μm); for the bacterial suspension used in the biocontrol treatment, the pellet was resuspended in sterile phosphate-buffered saline (PBS) and adjusted to 1 × 10^8^ CFU/mL. After centrifuging, the bacterial culture was reconstituted in sterile PBS and adjusted to a concentration of 1 × 10^8^ CFU/mL. The inoculation volume was standardized to 15 mL across all treatments. The pathogenic fungus inoculation method followed the procedure described in Section 2.2. Following pathogen inoculation, all pots were covered with transparent polyethylene sheets and maintained under high humidity (>90% RH) with electric humidifiers for 21 d to promote disease development. Disease severity was evaluated using the Standard Evaluation System for Rice (SES) published by the International Rice Research Institute (IRRI, 2013), ensuring consistency with established phenotypic evaluation protocols [43]. The incidence, severity index, and relative control efficacy were then calculated [44].

Incidence = (number of diseased leaves/total number of investigated leaves) × 100%.

Disease index = ∑(number of diseased leaves at all levels × disease progression)/(total number of surveyed leaves × highest disease progression) × 100%.

Relative control effect = (disease index of the control group − disease index of the treatment group)/disease index of the control group × 100%.

### 2.10. LC-MS Data Analysis

#### 2.10.1. Isolation and Purification of Antimicrobial Substances

The isolation and purification of antimicrobial substances from isolate 7-A were carried out with modifications based on the method of Gu [45]. In brief, isolate 7-A was cultured in 500 mL of Luria–Bertani broth (LB) at 28 °C with shaking at 180 rpm for 5 d. After incubation, the culture was centrifuged to collect the supernatant, which was then subjected to two sequential extractions with 250 mL of ethyl acetate over a total period of 8 h. The combined ethyl acetate phases were concentrated to dryness under reduced pressure with a rotary evaporator, yielding a yellow crude extract. This residue was subsequently dissolved in LC-MS-grade methanol for further analysis.

#### 2.10.2. LC-MS Analysis of Antimicrobial Metabolites

The chemical profiles of bioactive metabolites generated by isolate 7-A were analyzed using high-resolution liquid chromatography–mass spectrometry (LC-MS). The crude extract was diluted 10-fold and 100-fold with methanol. Each diluted sample was filtered through a 0.22 μm microporous membrane prior to analysis, then transferred into a dedicated autosampler vial for LC-MS analysis.

Chromatographic separation was performed on a Kinetex^®^ F5 column (100 mm × 2.1 mm, 2.6 μm), maintained at 40 °C. The mobile phases consisted of ultrapure water (phase A) and LC-MS-grade acetonitrile (phase B). A gradient elution program was applied as follows: 0–2 min, 5% B; 2–8 min, 5–60% B; 8–20 min, 60–95% B; 20–25 min, 95% B; 25–25.01 min, 95–5% B; and 25.01–27 min, 5% B. The flow rate was maintained at 300 μL/min, with an injection volume of 10 μL. UV detection was performed at 220 nm.

Mass spectrometric analysis was conducted using an electrospray ionization (ESI) source in both positive and negative ion modes. The ion spray voltage was set to 5000 V, and the ion source temperature was maintained at 500 °C. The mass scan range was set to m/z 100–1250. Data acquisition and processing were performed using SCIEX OS software (Version 1.7.0). The identified compounds were tentatively annotated by comparing their mass spectra and retention times with those reported in the literature [46,47].

### 2.11. Identification of Enzyme Activity Related to Plant Defense

In a controlled greenhouse setting, after inoculation with *F. verticillioides*, a total of three collections were conducted, and sampling was performed at the same anatomical position on the flag leaf sheath across all plants to minimize positional variability. The leaves were immediately stored at −80 °C for subsequent analytical procedures. In the leaves subjected to various interventions, the activity levels of several defense enzymes, including phenylalanine ammonia-lyase (PAL), peroxidase (POD), cathode (CAT), phenol oxidase (PPO), and superoxide dismutase (SOD), were assessed. The experimental design comprised three control groups: Healthy Control (HC) with plants treated only with Luria–Bertani broth (LB); Disease Control (DC) with plants inoculated solely with the pathogen; and Inducer Control (IC) with plants inoculated with *Bacillus* isolate 7-A without pathogen challenge. The treatment group (TR) involved sequential inoculation with isolate 7-A followed 24 h later by pathogen exposure to evaluate induced resistance. Defense enzyme activities—phenylalanine ammonia-lyase (PAL), peroxidase (POD), superoxide dismutase (SOD), polyphenol oxidase (PPO), and catalase (CAT)—were quantified using specified commercial colorimetric kits (Solarbio Science & Technology Co., Ltd., Beijing, China; catalog numbers: PAL/BC0210, POD/BC0090, SOD/BC0170, PPO/BC0190, and CAT/BC0200) per manufacturer protocols. Crude enzyme extracts were obtained by homogenizing plant tissues in chilled kit-specific extraction buffer, followed by centrifugation (12,000× *g*, 4 °C, 10 min); resulting supernatants were immediately analyzed spectrophotometrically by tracking absorbance changes at prescribed wavelengths and temperatures. All assays were conducted in triplicate, with enzyme activity expressed as units per gram fresh weight (U/g·FW) [46].

### 2.12. Statistical Analysis

Statistical analysis of the experimental data was conducted using SPSS 17.0 software (SPSS Inc., Chicago, IL, USA). Mean values were compared using Duncan’s Multiple Range Test at the 5% significance level (*p* < 0.05). Results are presented as means ± standard error of the mean (SEM).

## 3. Results

### 3.1. Pathogen Isolation and Morphological Identification

**Figure 1:**
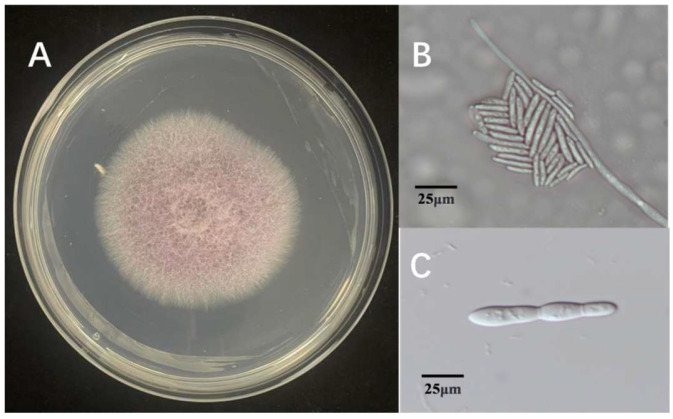
Morphological characteristics of *Fusarium* ZS97-1. (**A**): colony morphology on PDA medium; (**B**): microconidia observed under the microscope; (**C**): macroconidia observed under the microscope.

Three fungal isolates were found in tissue samples with symptoms. The isolates were determined to be members of the same fungal species based on morphological analysis, which showed little difference between them. Colonies with consistent morphology were observed on most isolation plates prepared from infected tissues. Every isolate thrived on PDA. The 90 mm Petri dishes were fully coated for 5 days. Colonies developed a light purple pigmentation with a cottony to fluffy surface texture, featuring smooth margins and slightly elevated central regions (Figure 1A). Under microscopic observation, hyphae ranged from hyaline to light brown in coloration. The isolate produced two distinct conidial types: microconidia, which were ellipsoidal to ovoid, hyaline to slightly pigmented, measured 3–5 × 1–2 μm (length × width), and typically formed dense clusters or false heads on short monophialides (Figure 1B); and macroconidia, which were fusiform, 3–5 septate, with straight to slightly curved dorsal contours and gradually tapered bases, measuring 30–50 × 4–6 μm (Figure 1C). These morphological characteristics, including colony appearance, hyphal pigmentation, and conidial morphology, are consistent with those of species within the genus *Fusarium*. Based on comprehensive morphological analysis, the causal agent of rice sheath rot in the Jingzhou region was preliminarily identified as a member of the genus *Fusarium*.

### 3.2. Molecular Identification of the Pathogenic Fungus

To accurately identify the fungal pathogen isolated from rice sheath rot, a phylogenetic analysis was performed based on sequence data of the internal transcribed spacer (ITS), *β*-tubulin (*TUB*), and the second largest subunit of RNA polymerase II (*rpb2*). The sequences generated were deposited in GenBank under accession numbers PV703760 (ITS), PV711425 (*TUB*), and PV711424 (*RPB2*). GenBank reference sequences were used for constructing a phylogenetic tree. The combined dataset was used to infer the taxonomic position of the isolate ZS97-1 in relation to other *Fusarium* species.

**Figure 2:**
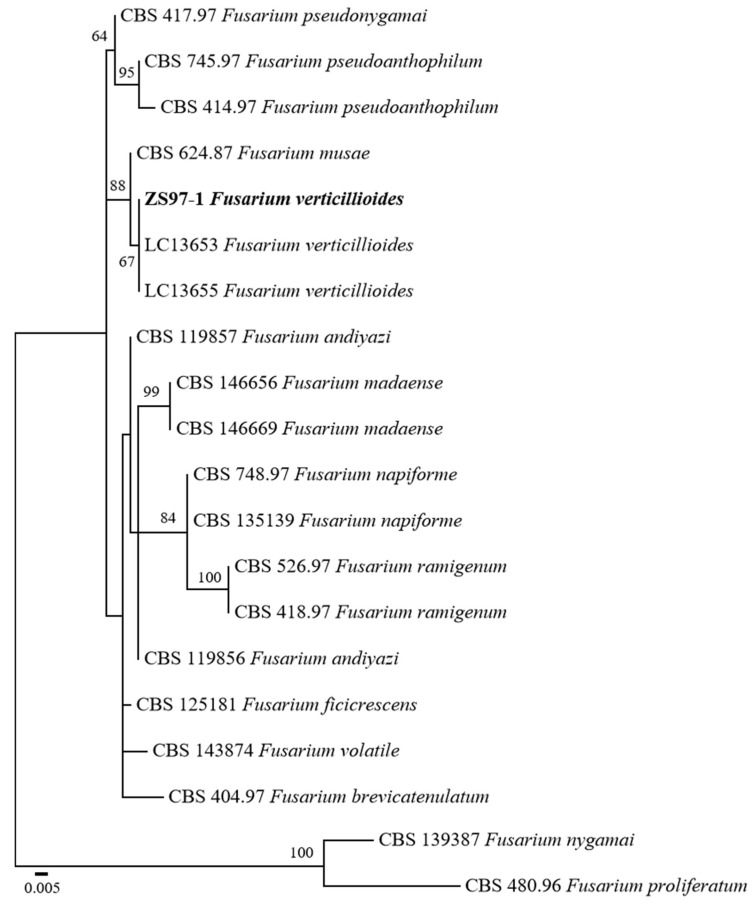
Maximum likelihood phylogenetic tree inferred from the combined ITS, *TUB*, and *RPB2* sequences of *Fusarium* species. Bootstrap values above 50% are indicated at the nodes. *F. nygamai* CBS 1,139,387 and *F. proliferatum* CBS 480.96 were used as outgroups. The strain isolated in this study is shown in bold. The scale bar represents 0.005 nucleotide substitutions per site.

A phylogenetic tree with maximum likelihood (ML) was constructed with 1000 bootstrap replicates. The resulting tree revealed that ZS97-1 clustered within the *F. verticillioides* clade (Figure 2). Notably, ZS97-1 formed a well-supported monophyletic group with two reference *F. verticillioides* isolates (LC13653 and LC13655), supported by a bootstrap value of 67%. This phylogenetic relationship strongly supports the molecular identification of ZS97-1 as *F. verticillioides*.

### 3.3. Results of Pathogenicity Assays

**Figure 3:**
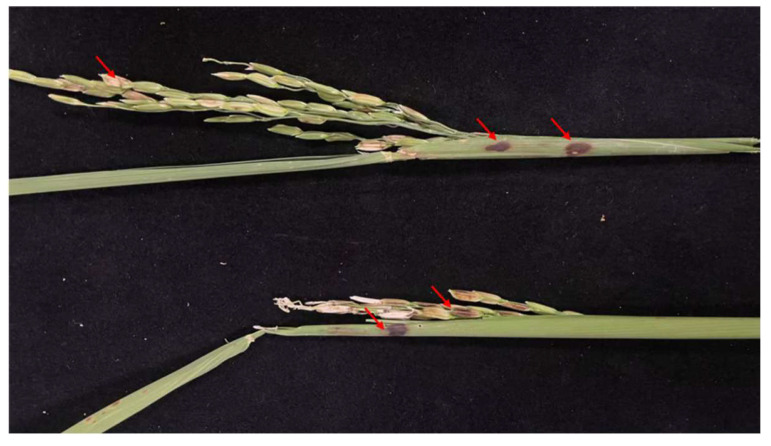
Pathogenicity of the isolated fungal isolate on inoculated rice plants. Arrows indicate the site of infection.

Rice plants inoculated with the Jingzhou isolate showed typical symptoms of sheath rot, characterized by distinct dark brown necrotic lesions on leaf sheaths, developed white panicles, and grain discoloration. These symptoms included both grain sterility (shriveled or empty seeds) and brown pigmentation of developed kernels (Figure 3). The re-isolated isolate exhibited morphological and cultural characteristics identical to those of the original inoculum, including colony appearance, pigmentation, and conidial morphology. These consistent traits fulfilled Koch’s postulates and confirmed the isolate’s etiological role in disease development. Based on these results, the Jingzhou isolate ZS97-1 was conclusively identified as a causal agent of rice sheath rot.

### 3.4. Morphological and Physiological Characterization of Isolate 7-A

**Figure 4:**
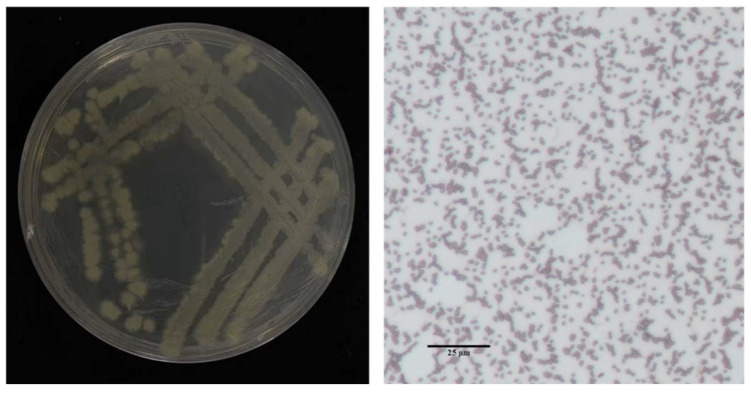
Colony morphology of isolate 7-A and Gram staining characteristics.

Eight bacterial isolates exhibiting inhibitory activity against the rice sheath rot pathogen were isolated from surface-sterilized tissues of healthy rice roots. Among these, isolate 7-A demonstrated significantly stronger antagonistic potential compared to the others and was selected for further characterization (Table 2). In LB medium, isolate 7-A formed milky white, opaque colonies with irregular (possibly serrated) margins on Luria–Bertani agar (LA) plates. Microscopic examination revealed short rod-shaped cells, consistent with typical *Bacillus* morphology (Figure 4).

**Figure 5:**
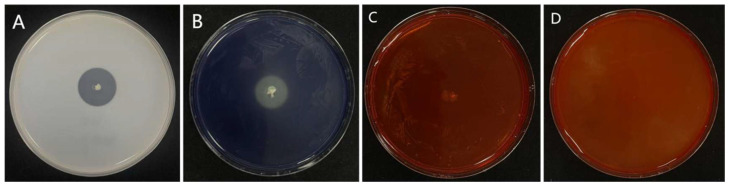
Production of extracellular enzymes by isolate 7-A. The isolate produced (**A**) protease, (**B**) siderophores, (**C**) cellulase, and (**D**) chitinase.

Extracellular enzyme profiling indicated that isolate 7-A produced protease and amylase, but showed no detectable activity for cellulase or chitinase (Figure 5). Further physiological and biochemical analyses revealed a distinct metabolic profile (Table 3): isolate 7-A metabolizes glucose via the glycolytic pathway, producing pyruvate as an intermediate. Pyruvate is subsequently converted into acetoin, detected via the Voges–Proskauer (V-P) test, and further metabolized into diacetyl and succinate. Additionally, the isolate can utilize citrate and D-mannitol as carbon sources, secrete proteases capable of degrading gelatin, reduce nitrate to nitrite or nitrogen gas, hydrolyze starch via amylase activity, and maintain metabolic activity under anaerobic conditions. In addition, isolate 7-A lacks the metabolic pathways required for the utilization of certain carbon sources and exhibits limited tolerance to high salinity. Gram staining confirmed that isolate 7-A is Gram-positive, further supporting its taxonomic placement. Taken together, the morphological, physiological, and biochemical characteristics strongly support the classification of isolate 7-A within the genus *Bacillus*.

### 3.5. Molecular Identification of Isolate 7-A

**Figure 6:**
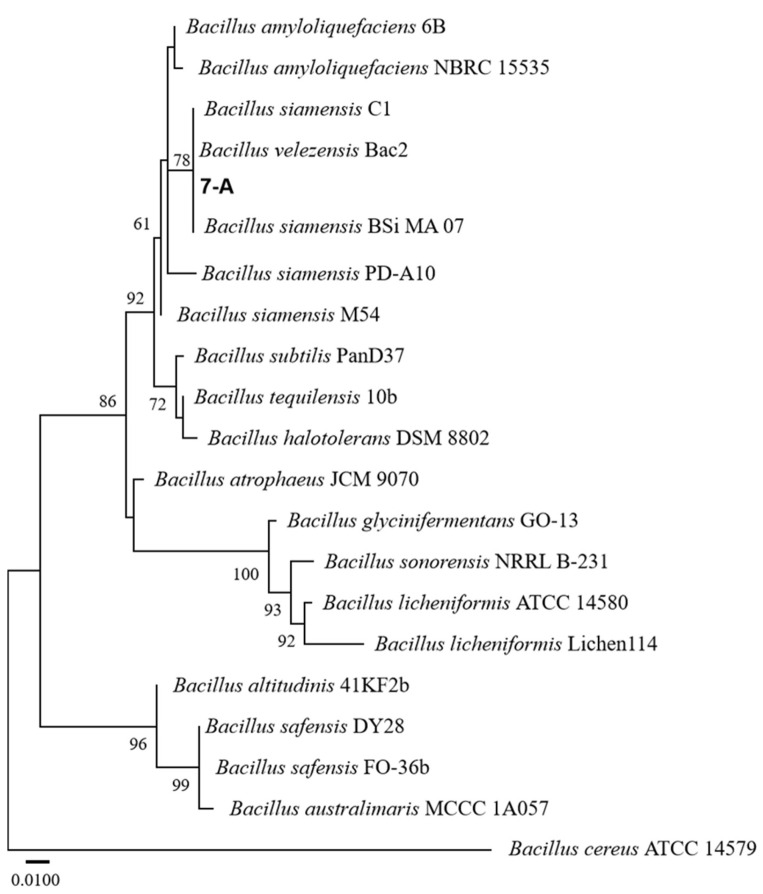
ML phylogenetic tree based on the concatenated 16S rRNA gene sequences of *Bacillus* species, with *Bacillus cereus* ATCC 14579 designated as the outgroup. The isolate isolated in this study is shown in bold. The scale bar represents 0.010 nucleotide substitutions per site.

**Figure 7:**
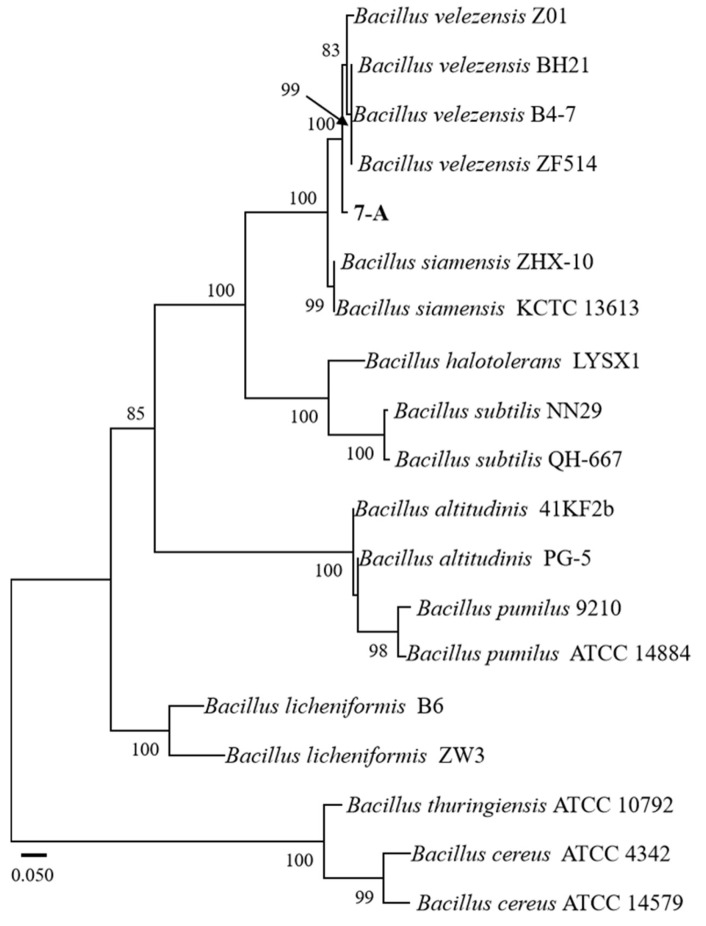
ML phylogenetic tree based on the combined sequences *gyrB* of *Bacillus* species. The isolate isolated in this study is shown in bold. The scale bar represents 0.050 nucleotide substitutions per site.

The sequence of isolate 7-A was deposited in GenBank, and accession numbers PV703759 (16S rRNA) and PV711423 (*gyrB*) were obtained. A phylogenetic tree was constructed using reference sequences retrieved from GenBank. Sequence identity analysis using BLAST (https://blast.ncbi.nlm.nih.gov/) revealed that both sequences exhibited 99% sequence similarity to those of *B. velezensis* in the GenBank database. In the phylogenetic trees constructed based on both the *gyrB* and 16S rRNA gene sequences, isolate 7-A clustered within the clade of *B. velezensis*, with bootstrap support values of 78% and 99%, respectively (Figure 6 and Figure 7). These results provide robust phylogenetic evidence supporting the molecular identification of isolate 7-A as *B. velezensis* at the species level.

### 3.6. Antimicrobial Spectrum of Isolate 7-A

**Figure 8:**
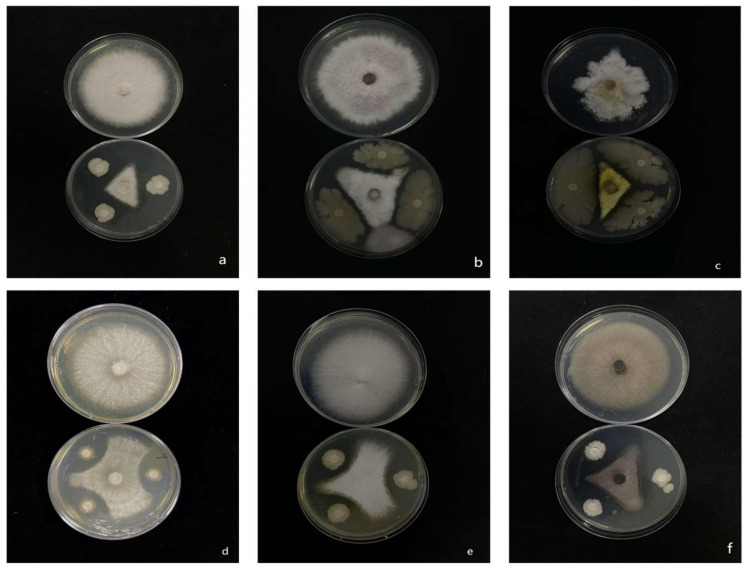
Inhibitory effect of isolate 7-A on the mycelial growth of six phytopathogenic fungi. (**a**): *Rigidoporus microporus*; (**b**): *F. oxysporum*; (**c**): *Cochliobolus heterostrophus*; (**d**): *Rhizoctonia solani*; (**e**): *F. fujikuroi*; (**f**): *Phytophthora nicotianae*.

Isolate 7-A exhibited pronounced antifungal activity, effectively inhibiting the mycelial growth of six economically important plant pathogens: *Rigidoporus microporus*, *F. oxysporum*, *Cochliobolus heterostrophus*, *Rhizoctonia solani*, *F. fujikuroi*, and *Phytophthora nicotianae* (Figure 8).

Quantitative analysis revealed that isolate 7-A consistently inhibited the growth of all tested pathogens by more than 50% (Table 4), underscoring its strong and broad-spectrum antifungal potential. The observed efficacy across phylogenetically diverse fungal and oomycete species highlights the isolate’s versatility as a biocontrol agent.

### 3.7. Inhibitory Effects of Isolate 7-A Bacterial Culture Filtrates (BCFs) on the Rice Sheath Rot Pathogen

**Figure 9:**
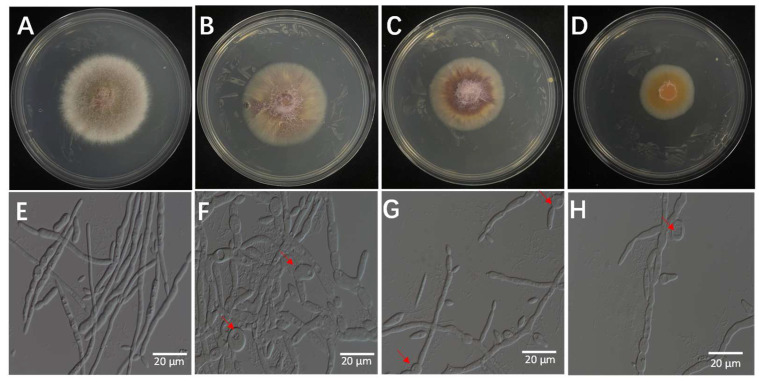
Effects of BCF on the mycelial growth of *F. verticillioides* at different concentrations: (**A**) (0%), (**B**) (1%), (**C**) (5%), and (**D**) (10%). Microscopic observation revealed structural abnormalities in the treated hyphae—(**F**) (1%), (**G**) (5%), and (**H**) (10%)—including twisting, swelling, and irregular branching, compared to the control ((**E**), 0%), which exhibited intact and normal hyphal morphology. The arrow indicates the site of hyphal swelling.

The bacterial culture filtrates (BCFs) obtained from the fermentation broth of isolate 7-A were evaluated for its antifungal activity against the rice sheath rot pathogen (*F. verticillioides* isolate ZS97-1) using a series of in vitro assays. As shown in Figure 9, the BCF exhibited dose-dependent inhibitory effects on mycelial growth of ZS97-1. Notably, microscopic observation revealed pronounced morphological alterations in fungal hyphae following BCF treatment. The mycelial biomass and colony diameter were significantly reduced in the treated groups, and the severity of pigment deposition increased with higher BCF concentrations (Figure 9A–D) (Supplementary Table S1).

Microscopic observation revealed extensive structural abnormalities in treated hyphae, including pronounced twisting and swelling. These morphological changes became more severe with increasing BCF concentration (Figure 9E–H), suggesting a concentration-responsive mode of action.

### 3.8. Pot Control Efficacy of Isolate 7-A Against Rice Sheath Rot

**Figure 10:**
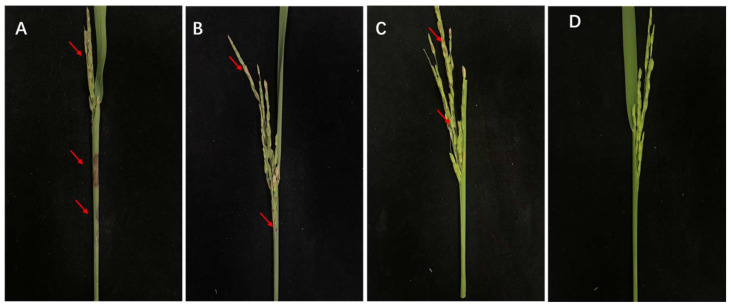
Control effect of isolate 7-A against rice sheath rot in potted rice plants. (**A**): plants inoculated solely with *F. verticillioides* without protective treatment; (**B**): plants pretreated with cell-free filtrate (BCF) of isolate 7-A followed by pathogen inoculation; (**C**): plants pretreated with 10% carbendazim water-dispersible granule (WDG, 1000-fold dilution) prior to pathogen inoculation; (**D**): plants treated with sterile suspension of isolate 7-A without pathogen challenge to evaluate plant responses to the bacterium alone. Arrows indicate typical disease lesions on the rice sheath and grain.

In the pot experiment, isolate 7-A (1 × 10^8^ CFU/mL) exhibited significant biocontrol activity against rice sheath rot (*F. verticillioides*). Negative control plants inoculated with the pathogen developed severe disease symptoms, characterized by extensive dark brown lesions on the leaf sheaths, white panicles, and grain discoloration, including both grain sterility (shriveled or empty seeds) and brown pigmentation of developed kernels (Figure 10A). In contrast, BCF-treated and positive-control plants showed markedly reduced symptom severity, with some exhibiting no visible infection(Figure 10B,C). Biocontrol treatment further confirmed that isolate 7-A was non-pathogenic to rice plants (Figure 10D), indicating its safety for application in agricultural systems. Both isolate 7-A and the 10% carbendazim water-dispersible granule (WDG) demonstrated strong inhibitory effects against rice sheath rot, resulting in disease indices of 4.94 and 4.12, respectively. Notably, the control efficacies reached 61.3% for isolate 7-A and 67.7% for 10% carbendazim water-dispersible granule (WDG) under pot conditions(Table 5).

### 3.9. LC-MS Analysis of the Sterile Fermentation Broth of Isolate 7-A

**Figure 11:**
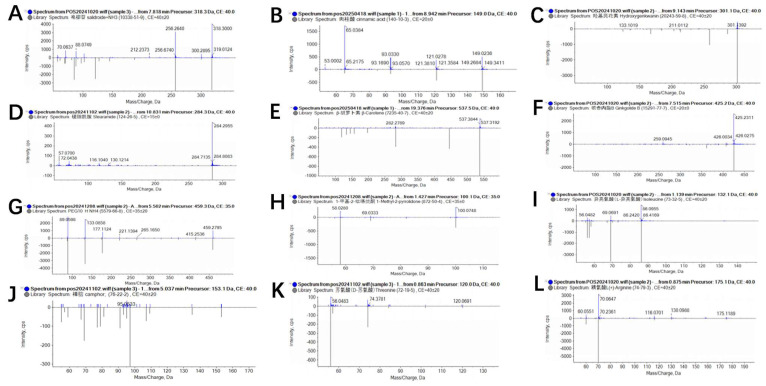
Mass spectrometric analysis of lipopeptides and other bioactive compounds produced by isolate 7-A. Detected metabolites included the following: (**A**), cinnamic acid; (**B**), salicin; (**C**), hydroxygenkwanin; (**D**), stearamide; (**E**), *β*-carotene; (**F**), ginkgolide B; (**G**), 1-methyl-2-pyrrolidone; (**H**), camphor; (**I**), PEG-10 hydrogenated ammonium; (**J**), threonine; (**K**), arginine; and (**L**), isoleucine.

Liquid chromatography–mass spectrometry (LC-MS) analysis of the crude extract from isolate 7-A led to the identification of multiple chemical constituents, including amino acids, organic acids, glycosides, terpenoids, and lipophilic compounds. These metabolites exhibited distinct retention times and high mass accuracy, indicating a diverse metabolic profile (Figure 11). Notably, hydroxygenkwanin, cinnamic acid, and *β*-carotene were among the predominant components, suggesting that isolate 7-A possesses the potential for biosynthesis of aromatic and carotenoid-type compounds. The complete list of identified compounds and their relative abundances is presented in Table 6.

### 3.10. Impact of Isolate 7-A on Rice Defense-Related Enzyme Activity

**Figure 12:**
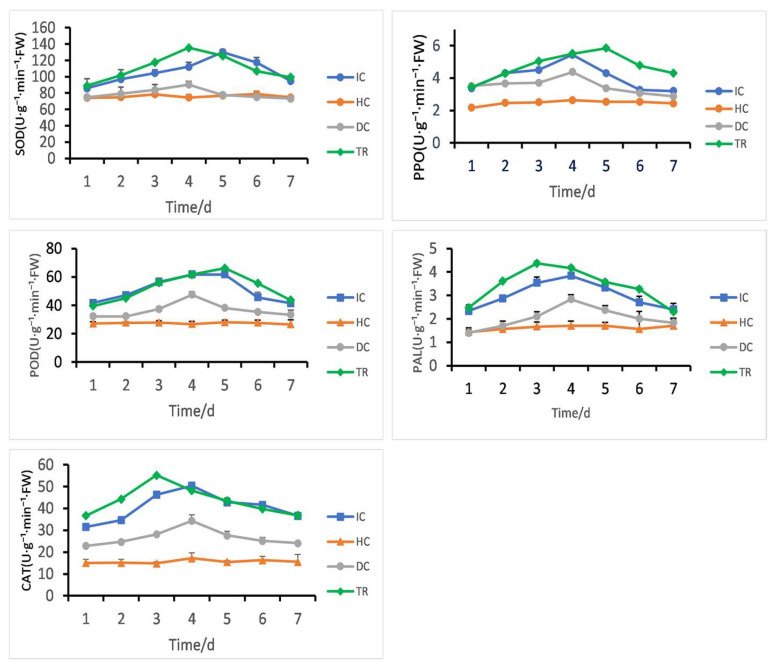
*B. velezensis* 7-A induces defense enzyme activities (PPO, SOD, PAL, CAT, and POD) in rice under different treatments: Healthy Control (HC)—plants treated with Luria–Bertani broth without biocontrol agent or pathogen; Disease Control (DC)—plants inoculated with pathogen only; Inducer Control (IC)—plants inoculated with *B. velezensis* 7-A without pathogen challenge; and Treatment (TR)—plants first inoculated with *B. velezensis* 7-A followed by pathogen inoculation 24 h later to assess induced resistance.

Following different treatment groups, Figure 12 illustrates the variations in the activities of POD, PPO, PAL, CAT, and SOD in rice leaves. The activities of key defense-related enzymes were significantly induced in the treated group (TR) compared to controls (HC, IC, DC).

Catalase (CAT) and phenylalanine ammonia-lyase (PAL) activities peaked at day 3, with CAT reaching 55.2 U·g^−1^·min^−1^·FW and PAL 4.3 U·g^−1^·min^−1^·FW. Peroxidase (POD) and polyphenol oxidase (PPO) also increased, showing maximum activity at day 5 (66.1 U·g^−1^·min^−1^·FW for POD, 5.8 U·g^−1^·min^−1^·FW for PPO). Superoxide dismutase (SOD) exhibited an early peak at day 4 (135.4 U·g^−1^·min^−1^·FW), followed by a gradual decline.

In contrast, enzyme activities in the Healthy Control (HC) and Disease Control (DC) groups remained low throughout the experiment. The inoculated control (IC) showed only minor fluctuations.

These results indicate that the TR treatment activated a time-dependent defense response, with coordinated upregulation of antioxidant and phenylpropanoid pathway enzymes, peaking between days 3 and 5.

## 4. Discussion

Rice sheath rot is an increasingly significant constraint to rice production worldwide, especially in high-input agricultural systems, where favorable environmental conditions and intensive management practices promote disease development [3]. In this study, we identified *F*. *verticillioides* as a pathogen associated with rice sheath rot, which is not commonly recognized as a primary causal agent of the disease under field conditions. The isolate 7-A was identified as *B. velezensis*, a potent biocontrol agent against *F. verticillioides*, which represents a significant advancement in the development of sustainable alternatives to chemical fungicides. Isolate 7-A exhibited strong antifungal activity, achieving high mycelial growth inhibition in vitro and demonstrating broad-spectrum efficacy against multiple phytopathogens, including *R. solani*, *C*. *heterostrophus*, and *F*. *oxysporum*. These results are in agreement with previous studies that have demonstrated the multi-target antagonistic potential of *B. velezensis* through the production of diverse secondary metabolites, such as lipopeptides, polyketides, and antimicrobial peptides [48].

In this study, isolate 7-A does not secrete chitinase, amylase, or cellulase, but it produces proteases. Additionally, isolate 7-A can release siderophores. In addition, siderophores produced by beneficial microorganisms are crucial for defending plants against fungal diseases [49]. These tiny organic molecules have a strong affinity for Fe^3+^, and can prevent the formation of dangerous fungi by keeping iron levels below ideal for regular metabolic processes [50].

Notably, the BCF of isolate 7-A induced significant morphological alterations in *F. verticillioides*, including hyphal swelling and structural deformation, so we suggest that the antifungal activity is likely mediated by diffusible bioactive compounds; this observation is consistent with previous studies demonstrating that *Bacillus* species primarily exert antifungal activity through the secretion of extracellular antimicrobial compounds, which target fungal cell wall integrity and membrane stability [51].

In the pot experiment, the BCF of isolate 7-A demonstrated a significant disease control potential, achieving disease suppression efficacy comparable to disease control efficacy of the synthetic fungicide carbendazim. According to Qin et al. [52], greenhouse trials demonstrated that the B5 fermentation broth successfully prevented tobacco brown spot disease, attaining a relative control efficacy of 60.66%, which is equivalent to 10% difenoconazole water-dispersible granule (WDG); this aligns with our experimental findings. Our results underscore the practical potential of isolate 7-A as a biocontrol agent under controlled conditions and provide a foundation for future field evaluations. Moreover, the absence of phytotoxic effects during the pot experiments indicate its compatibility with rice plants, further supporting its suitability for integrated disease management programs.

Secondary metabolite profiling revealed that isolate 7-A produces a variety of bioactive compounds: threonine, arginine, isoleucine, 1-Methyl-2-pyrrolidone, camphor, PEG-10 hydrogenated ammonium, ginkgolides B, salicin, cinnamic acid, hydroxygenkwanin, stearamide, and *β*-carotene, most of which have been previously reported to possess antimicrobial, antioxidant, anti-inflammatory, and signaling-modulating activities. According to Kong et al. [53] and Bisognoet et al. [54], camphor and cinnamic acid exhibit promising antifungal activity, which is attributed to their ability to disrupt the cell membrane integrity of fungal pathogens and interfere with essential metabolic processes, thereby inhibiting or eliminating fungal growth. Amino acids such as arginine may enhance plant disease resistance by inducing nitric oxide (NO) production, which in turn activates systemic acquired resistance (SAR) [55]. Previous studies have demonstrated that *β*-carotene, ginkgolide B, and salicin demonstrated strong antioxidant properties, which can help alleviate oxidative stress for plants under adverse conditions, and maintain physiological homeostasis, indirectly improving plant resilience to pathogens [56,57,58]. Notably, previous studies have reported that PEG-10 hydrogenated ammonium and 1-methyl-2-pyrrolidone (NMP) can function as formulation adjuvants [59,60]. These excipients play a critical role in stabilizing active pharmaceutical ingredients (APIs) and enhancing their solubility, consequently improving the bioavailability of the formulations. The coexistence of multiple functional molecules suggests that isolate 7-A may employ a synergistic mode of action, targeting different physiological processes in the pathogen and thereby reducing the likelihood of resistance development. The multi-target mechanisms are highly advantageous in biocontrol agents, as they enhance both the spectrum and sustainability of disease suppression.

Enzymes involved in plant defense can neutralize reactive oxygen species and activate the signaling pathways of salicylic acid (SA), jasmonic acid (JA), and ethylene (ET) to enhance plant resistance to pathogen infection [61,62,63]. Plants activate defense enzymes such as PPO, SOD, PAL, CAT, and POD in response to exposure to various pathogenic organisms. These enzymes are essential for the metabolism of ROS [64,65]. The production of secondary metabolites, such as lignin, phenolic compounds, and defense hormones, plays a crucial role in plant disease resistance [66], and they directly inhibit and kill pathogenic bacteria, thereby enhancing plant resistance [67]. Several studies have shown a positive correlation between the disease resistance index and the activity of enzymes like PPO, SOD, PAL, CAT, and POD, indicating a specific defense mechanism against infections. In this study, isolate 7-A significantly increased the activity of several defense-related enzymes in rice plants, with the effect becoming more pronounced as the concentration increased. These results are in agreement with the findings reported by Qiu et al. [62]. Comparable biocontrol activity has been documented for *B. velezensis* SDTB022, a tomato rhizosphere strain, while our research demonstrates similar efficacy with isolate 7-A.

The ecological adaptability and rhizosphere competence of *B. velezensis* make it suitable for application in sustainable agriculture. Previous studies have shown that strains of these species can suppress the pathogens directly and induce systemic resistance (ISR) in host plants and promote plant growth through the synthesis of phytohormones and other growth regulators [68].

The aforementioned findings suggest that the biocontrol activity of isolate 7-A against rice sheath rot may be associated with multiple mechanisms and their combined effects, which means that it holds strong promise as a candidate for biological control of rice sheath rot. However, several key areas require further investigation before large-scale field deployment can be considered. These include determining the components of 7-A’s antifungal chemicals, analyzing the expression patterns of defense-related marker genes using qPCR or other transcriptomic approaches, and validating its efficacy under natural field conditions.

## 5. Conclusions

In summary, this study aims to isolate and identify *F*. *verticillioides* as an infrequent causal agent of rice sheath rot, and systematically evaluates the biocontrol potential of *B. velezensis* isolate 7-A (endophytic bacteria in rice roots) against rice sheath rot. Furthermore, by combining conventional microbiological methods with advanced molecular and metabolic analyses, this work provides a basis for the development of sustainable, biocontrol-based strategies for the management of this significant rice disease.

## Figures and Tables

**Table 2:** Antimicrobial activity of isolates against *F. verticillioides*.

Isolated	Inhibition Zone
7-A	+++
7-B	+
7-C	++
7-D	+
7-E	+
7-F	+
7-G	+
7-H	++

Note: the diameter of the inhibition zone reflects the antimicrobial efficacy of each isolate. Weak (+): <10 mm; moderate (++): 10–15 mm; strong (+++): >15 mm.

**Table 3:** Physiological and biochemical characteristics of isolate 7-A.

Test Parameter	Result
Voges–Proskauer test	+
Citrate utilization	+
Propionate utilization	−
D-Xylose fermentation	−
L-Arabinose fermentation	−
D-Mannitol fermentation	+
Gelatin liquefaction	+
Growth in 7% NaCl	−
Nitrate reduction	−
Starch hydrolysis	+
Anaerobic growth	+
Voges–Proskauer test	+

Note: “+” denotes positive results, “−” indicates negative results.

**Table 4:** Antimicrobial spectrum of *Bacillus velezensis* 7-A.

Pathogen	Disease	Relative Inhibition Rate (%)
*Rigidoporus microporus*	Red root disease in rubber trees	70.0 ± 2.6 a
*Fusarium oxysporum*	*Fusarium* wilt in eggplant	63.6 ± 2.4 b
*Cochliobolus heterostrophus*	Southern corn leaf blight	68.9 ± 2.2 a
*Rhizoctonia solani*	Rice sheath blight	54.5 ± 1.2 d
*Fusarium fujikuroi*	Rice bakanae disease	58.3 ± 0.7 c
*Phytophthora nicotianae*	Target spot in tobacco	71.8 ± 1.6 a

The values provided represent the mean ± SD of three replicates. Values followed by the same letter are not statistically significantly different at a *p*-value threshold of ≤0.05, as determined by Duncan’s Multiple Range Test.

**Table 5:** Control efficacy of isolate 7-A against rice sheath rot disease in pot experiments.

Treatment	Disease Index	Control Effect (%)
*F. verticillioides*	12.76 ± 0.58 b	--
*F. verticillioides +* 7-A	4.94 ± 1.01 a	61.3 ± 1.7 b
*F. verticillioides +* 10% carbendazim	4.12 ± 1.16 a	67.7 ± 1.2 a

The values provided represent the mean ± SD of three replicates. Values followed by the same letter are not statistically significantly different at a *p*-value threshold of ≤0.05, as determined by Duncan’s Multiple Range Test.

**Table 6:** LC- MS analysis of components in the crude extract of isolate 7-A.

Retention Time (min)	Mass–Charge Ratio (*m*/*z*)	Ion Binding Mode	Substance Type	Relative Peak Area	Elative Ratio (%)
0.86	120.0026	[M + H]+	Threonine	2.615 × 10^3^	0.56
0.88	175.1188	[M + H]+	Arginine	2.146 × 10^4^	4.56
1.14	132.1013	[M + H]+	Isoleucine	1.452 × 10^4^	3.08
1.43	100.0750	[M + H]+	1-Methyl-2-pyrrolidone	4.383 × 10^3^	0.93
5.04	153.0654	[M + Na]+	Camphor	3.037 × 10^4^	6.45
5.50	686.7601	[M + H]+	PEG-10 Hydrogenated Ammonium	1.805 × 10^3^	0.38
7.52	425.2300	[M + H]+	Ginkgolides B	1.593 × 10^3^	0.34
7.82	149.0230	[M + 3H3+]+	Salicin	1.033 × 10^4^	2.19
8.94	301.1408	[M + H]+	Cinnamic Acid	5.427 × 10^4^	11.53
9.14	284.3316	[M + H]+	Hydroxygenkwanin	2.742 × 10^5^	58.24
10.83	148.7771	[M + H]+	Stearamide	1.434 × 10^4^	3.05
19.38	284.9959	[M + H]+	*β*-carotene	4.095 × 10^4^	8.70

## Data Availability

The data underlying the findings are not publicly available due to restrictions from a third-party agreement and commercial confidentiality. Access may be granted by the corresponding authors upon reasonable request and with approval from the involved partner.

## Supplementary Materials

The following supporting information can be downloaded at: https://www.mdpi.com/article/10.3390/microorganisms13112511/s1, Table S1: Antifungal activity of different concentrations of 7-A BCF in plate assay. File S1: Sequence alignment of strain ZS97-1 based on *TUB*, *rpb2*, and ITS regions. File S2: 16S rRNA gene sequence alignment of strain 7-A. File S3: *gyrB* rRNA gene sequence alignment of strain 7-A.
