# Supplementary material for: Bacillus velezensis 7-A as a Biocontrol Agent Against Fusarium verticillioides, the Causal Agent of Rice Sheath Rot Disease
Source: Microorganisms. 2025 Oct 31;13(11):2511. doi: 10.3390/microorganisms13112511 (PMC12654073; doi:10.3390/microorganisms13112511)
Supplement: Supplementary file 1 [file microorganisms-13-02511-s001.zip › Supplementary Table S1.pdf]

Supplementary Table S1. The inhibitory effect of different concentrations of sterile filtrate on *Fusarium verticillioides* was assessed by growing the pathogen on PDA medium incorporated with culture filtrates for 2 d, after which colony diameters were measured.

Filtrate Concentration	Colony diameters (mm)	Inhibition rate(%)
0%	33.23±0.55d	--
1%	28.30±0.97c	15c
5%	25.94±0.72b	21b
10%	17.60±1.24a	47a
